# Association of *HMOX1* and *NQO1* Polymorphisms with Metabolic Syndrome Components

**DOI:** 10.1371/journal.pone.0123313

**Published:** 2015-05-01

**Authors:** Angélica Martínez-Hernández, Emilio J. Córdova, Oscar Rosillo-Salazar, Humberto García-Ortíz, Cecilia Contreras-Cubas, Sergio Islas-Andrade, Cristina Revilla-Monsalve, Consuelo Salas-Labadía, Lorena Orozco

**Affiliations:** 1 Immunogenomics and Metabolic Disease Laboratory, Instituto Nacional de Medicina Genómica, SS, Mexico City, Mexico; 2 Medical research unit in Metabolic Disease, Centro Médico Nacional Siglo XXI, IMSS, Mexico City, Mexico; 3 Tissue culture Laboratory, Human Genetic Department, Instituto Nacional de Pediatría, SS, Mexico City, Mexico; University of Catania, ITALY

## Abstract

Metabolic syndrome (MetS) is among the most important public health problems worldwide, and is recognized as a major risk factor for various illnesses, including type 2 diabetes mellitus, obesity, and cardiovascular diseases. Recently, oxidative stress has been suggested as part of MetS aetiology. The heme oxygenase 1 (*HMOX1*) and NADH:quinone oxidoreductase 1 (*NQO1*) genes are crucial mediators of cellular defence against oxidative stress. In the present study, we analysed the associations of *HMOX1* (GT)*n* and *NQO1* C609T polymorphisms with MetS and its components. Our study population comprised 735 Mexican Mestizos unrelated volunteers recruited from different tertiary health institutions from Mexico City. In order to know the *HMOX1* (GT)*n* and *NQO1* C609T allele frequencies in Amerindians, we included a population of 241 Amerindian native speakers. Their clinical and demographic data were recorded. The *HMOX1* (GT)*n* polymorphism was genotyped using PCR and fluorescence technology. *NQO1* C609T polymorphism genotyping was performed using TaqMan probes. Short allele (<25 GT repeats) of the *HMOX1* polymorphism was associated with high systolic and diastolic blood pressure, and the T allele of the *NQO1* C609T polymorphism was associated with increased triglyceride levels and decreased HDL-c levels, but only in individuals with MetS. This is the first study to analyse the association between MetS and genes involved in oxidative stress among Mexican Mestizos. Our data suggest that polymorphisms of *HMOX1* and *NQO1* genes are associated with a high risk of metabolic disorders, including high systolic and diastolic blood pressure, hypertriglyceridemia, and low HDL-c levels in Mexican Mestizo individuals.

## Introduction

Metabolic syndrome (MetS) is a major public health problem in Mexico, where the prevalence (36.8%) is among the highest worldwide [1]. The National Cholesterol Education Program Adult Treatment Panel III (NCEP ATP III) describes MetS as including multiple metabolic abnormalities that are major risk factors for cardiovascular diseases (CVD) and type 2 diabetes, including abdominal obesity (high waist circumference), hyperglycaemia, hypertriglyceridemia, hypertension, and low levels of high-density lipoprotein-cholesterol (HDL-c) [2].

Oxidative stress—an abnormal accumulation of reactive oxygen species (ROS)—plays an important role in MetS aetiopathology [3]. Patients with MetS show increased oxidative stress and decreased concentrations of the antioxidants vitamins C and E and **β**-carotenoids [4–6]. Enhanced ROS generation has been observed in mouse models of obesity (*ob/ob)* and MetS. Moreover, the diet-derived antioxidant resveratrol reportedly improves glucose tolerance, and plasma levels of insulin and triglycerides [4,7,8].

The Keap1/Nrf2/ARE pathway plays a central role in regulating the expressions of genes that are crucial mediators of cellular defence against oxidative stress and chronic inflammation such as the *HMOX1* gene that encodes heme oxygenase 1 (HO-1) and the *NQO1* gene that encodes NADPH-quinone oxidoreductase 1 [9,10]. Polymorphisms in these genes can affect their antioxidant functions. For instance, a di-nucleotide repeat polymorphism located at the promoter region of the *HMOX1* gene modulates HO-1 expression levels [11], and the C609T single-nucleotide polymorphism (SNP) in *NQO1* causes a proline-to-serine amino acid change that results in loss of NQO1 activity in T homozygous humans [12,13]. Association studies have shown that these polymorphisms are involved in different oxidative stress-related diseases, such as cancer, diabetes, and Alzheimer’s disease [9,14–17]. Despite the important role of oxidative stress in metabolic diseases, the associations of *HMOX1* and *NQO1* with MetS have not yet been thoroughly studied.

The present study aimed to investigate the associations between *HMOX1 a*nd *NQO1* polymorphisms and MetS in Mexican individuals.

## Material and Methods

This study was performed in accordance with the Declaration of Helsinki, and was approved by the local ethics and research committees of the National Institute of Genomic Medicine. All participants signed informed written consent. This study included 735 Mexican Mestizo unrelated volunteers, all of them recruited from tertiary level institutions located in Mexico City. Demographic (age, sex and place of birth) and anthropometric (weight, size, waist circumference and blood pressure) data were collected. The waist circumference was measured at the midpoint between the last rib and the iliac crest, whereas body weight and body mass index (BMI) were determined using an electronic digital scale (Tanita Body Composition Analyzer, Model TBF-215), and was calculated as weight in kilograms/height in meters squared. Systolic and diastolic blood pressure was measured three times in the sitting position after resting for at least 5 min using an automatic electronic sphygmomanometer, according with the Mexican Official Standard (NOM-030-1999-SSA2). All biochemical data (glucose, triglyceride and HDL-C) were measured in all participants in a fasting state with Synchron CX5 Analyzer System (Beckman Coulter, Fullerton, CA). According to the criteria of the Cholesterol Education Program (NCEP) ATP III, MetS diagnosis was established when three or more of the following risk factors were identified: average of blood pressure ≥130/≥85 mmHg, glucose ≥100 mg/dL, triglycerides ≥150 mg/dL, HDL-c <40 in men and <50 mg/dL in women, and waist circumference >102 cm in men and >88 cm in women. In order to know frequencies of the risk alleles in Mexican-Amerindians, we included a population of 241 unrelated Amerindians from different regions of Mexico, these subjects born in their communities and speak their native language.

Genomic DNA was isolated from whole blood samples containing EDTA using the QIAamp DNA Blood Maxi kit (Qiagen, Valencia CA). The *HMOX1* (GT)*n* polymorphism was genotyped using a fluorescent-labelled forward primer (FAM 5'-GCTCTGGAAGGAGCAA AATCACACC-3') and an unlabelled reverse primer (5'-TATGACCCTTGGGAAACAAAG TCTGG-3'). PCR product sizes were determined using an automated DNA capillary sequencer with a molecular weight standard (GeneScan-500 LIZ size standard, Applied Biosystems, Foster City, CA) and the Peak Scanner software v1.0 (FAL3730xl DNA Analyzer; Applied Biosystems, Foster City, CA). The number of (GT)*n* repeats was determined using a pGL3 vector containing a 30-repeat fragment as a reference. Based on the number of GT repeats, we categorized samples as L allele (≥25 repeats) or S allele (<25 repeats). Allelic discrimination of the *NQO1* C609T SNP was performed by TaqMan genotyping assay on an ABI Prism 7900HT Fast Real-Time PCR system (Applied Biosystems, Foster City, CA, USA). To confirm the *HMOX1* or *NQO1* genotype, a randomly selected 10% of samples were directly sequenced.

Statistical analysis were performed using PLINK software [18] and SPSS v20.0 (SPSS, Chicago, IL, USA). Logistic regression models were used to evaluate the effect of having a risk allele on MetS or its components, with adjustment for body mass index (BMI), age, gender, and medication for diabetes, hypertension, or dyslipidaemic traits. The components of MetS were also analysed as a continuous trait utilizing linear regression models. Calculations of odds ratio (OR) with 95% confidence intervals (95% CI) and the Hardy–Weinberg equilibrium (HWE) were performed using FINETTI software (http://ihg.gsf.de/cgi-bin/hw/hwa1.pl). Statistical power was computed using a web browser program, Genetic Power Calculator [18] (http://pngu.mgh.harvard.edu/~purcell/gpc/). We conducted this calculation under various assumptions about genetic models (i.e., allelic, additive, dominant, recessive, and co-dominant models), assuming a minor allele frequencies (MAFs) of 16%, a disease prevalence of 45%, case-to-control of 1:1. The level of statistical significance was defined as *P* values of ≤0.05 after Bonferroni correction.

## Results

In Mexican Mestizo population, 65.7% were women and 34.3% were men, and the average age was 43.9 ± 7.6 years. The most common MetS component was low HDL-c (78%), followed by elevated glucose levels (61.4%), and the least common component was hypertension (19%) (Table 1).

**Table 1:** Characteristics of the Mexican Mestizo population.

Women	65.7%
Male	34.3%
Age (years)	43.9 ± 7.6
Triglycerides (≥150 mg/dL)	61.1%
Glucose (≥100 mg/dL)	61.4%
HDL-c	78%
Waist circumference	47.2%
Blood Pressure (≥130/≥85 mmHg)	19%

Age = average ± standard deviation.

HDL-c (< 40 men or < 50 mg/dL women),

Waist circumference (> 102 cm men or > 88 cm women).

The genotype distributions of *HMOX1* (GT)*n* and *NQO1* C609T polymorphisms were in Hardy–Weinberg equilibrium among the cases and the controls. The Mestizo population included 420 individuals with MetS (57%) and 315 controls (43%). Comparing the distributions of allele and genotype frequencies of *HMOX1* (GT)*n* and *NQO1* C609T polymorphisms did not reveal significant differences between cases and controls, even after stratification by gender, age, BMI and medicament status (Tables 2 and 3).

**Table 2:** Genotype and allele frequencies of *HMOX1* (GT)*n* polymorphism in Mexican Mestizo individuals.

Genotype/Allele	MetS	Control	OR	CI	*P* value
**Total population**					
	n = 420	n = 315			
**LL**	0.7 (296)	0.71 (223)			
**SL**	0.26 (109)	0.25 (79)	0.9	0.4–1.9	1.4
**SS**	0.04 (15)	0.04 (13)	1.0	0.7–1.5	1.6
**L**	0.83 (701)	0.83 (525)			
**S**	0.17 (139)	0.17 (105)	1.0	0.8–1.3	1.8
**Female**					
	n = 258	n = 225			
**LL**	0.71 (184)	0.69 (156)			
**SL**	0.27 (70)	0.26 (59)	1.0	0.7–1.5	1.9
**SS**	0.02 (4)	0.04 (10)	0.3	0.1–1.3	0.1
**L**	0.85 (438)	0.82 (371)			
**S**	0.15 (78)	0.18 (79)	0.8	0.6–1.2	0.6
**Male**					
	n = 162	n = 90			
**LL**	112 (0.69)	67 (0.74)			
**SL**	39 (0.24)	19 (0.21)	1.2	0.7–2.3	1.0
**SS**	11 (0.07)	4 (0.04)	1.6	0.5–5.4	1.4
**L**	263 (0.81)	153 (0.85)			
**S**	61 (0.19)	27 (0.15)	1.3	0.8–2.2	0.5

MetS = Metabolic syndrome. Data are presented as frequency (sample size).

*P* values were adjusted by age, BMI, gender, medicament status,

and Bonferroni correction.

**Table 3:** Genotype and allele frequencies of *NQO1* C609T polymorphism in Mexican Mestizo individuals.

Genotype/ Allele	MetS	Control	OR	CI	*P* value
**Total population**					
	n = 420	n = 315			
**CC**	0.31 (129)	0.27 (86)			
**CT**	0.53 (221)	0.52 (164)	0.9	0.6–1.3	1.1
**TT**	0.17 (70)	0.21 (65)	0.7	0.5–1.1	0.3
**C**	0.57 (479)	0.53 (336)			
**T**	0.43 (361)	0.47 (294)	0.9	0.7–1.1	0.3
**Female**					
	n = 258	n = 225			
**CC**	0.33 (84)	0.3 (67)			
**CT**	0.5 (130)	0.48 (108)	1.0	0.6–1.4	1.7
**TT**	0.17 (44)	0.22 (50)	0.7	0.4–1.2	0.3
**C**	0.58 (298)	0.54 (242)			
**T**	0.42 (218)	0.46 (208)	0.8	0.7–1.1	0.4
**Male**					
	n = 162	n = 90			
**CC**	0.28 (45)	0.21 (19)			
**CT**	0.56 (91)	0.62 (56)	0.7	0.4–1.3	0.5
**TT**	0.16 (26)	0.17 (15)	0.7	0.3–1.7	0.9
**C**	0.56 (181)	0.52 (94)			
**T**	0.44 (143)	0.48 (86)	0.9	0.6–1.2	0.9

MetS = Metabolic syndrome. Data are presented as frequency (sample size).

*P* values were adjusted by age, BMI, gender, medicament status,

Bonferroni correction.

To investigate the associations of *HMOX1* (GT)*n* and *NQO1* C609T polymorphisms with MetS components, we stratified each component according to the available reference value. We analysed participants with MetS first, and then the whole group (MetS plus control individuals).

We observed that neither the *HMOX1* (GT)*n* nor *NQO1* C609T polymorphisms were associated with MetS, however, both *HMOX1 and NQO1* were associated with some of its components.

Either in whole population and in MetS group, we observed a significant association between the *HMOX1* S allele and high blood pressure, which remained significant after adjustment for age, gender, and BMI. Both associations showed an additive effect, with a relative risk observed 2-fold higher for the homozygote risk genotype than that observed for the single risk allele (Table 4). Quantitative analysis revealed significant systolic pressure beta value was 3.2 (*P* = 0.009) in the MetS group, however in the whole population a significant trend was observed (*P* = 0.07). The diastolic pressure beta values were in the whole population (1.3) and in the MetS group (1.7). The *HMOX1* (GT)*n* repeat was not associated with any other MetS component (Table 4).

**Table 4:** Associations of *HMOX1* (GT)*n* polymorphisms with components of metabolic syndrome.

	All Population			Metabolic Syndrome		
	Below	Above	OR; *P*	Below	Above	OR; *P*
**Glucose**						
	n = 284	n = 451		n = 52	n = 368	
**SL**	0.24	0.26	1.1; 1.0	0.21	0.27	1.4; 0.6
**SS**	0.04	0.04	1.2; 1.2	0	0.04	5.0; 0.2
**S**	0.16	0.17	1.1; 0.8	0.11	0.17	1.8; 0.2
**HDL-C**						
	n = 573	n = 162		n = 389	n = 31	
**SL**	0.25	0.27	0.9; 1.4	0.25	0.35	0.6; 0.3
**SS**	0.04	0.04	0.9; 1.4	0.03	0.06	2.4; 0.5
**S**	0.16	0.18	0.9; 1.2	0.16	0.24	0.6; 0.2
**Triglycerides**						
	n = 286	n = 449		n = 73	n = 347	
**SL**	0.27	0.25	0.9; 1.1	0.26	0.26	1.0; 1.9
**SS**	0.04	0.04	0.9; 1.4	0.03	0.04	1.4; 1.3
**S**	0.17	0.16	0.9; 1.0	0.16	0.17	1.1; 1.5
**Waist Circumference**						
	n = 388	n = 347		n = 143	n = 277	
**SL**	0.26	0.24	0.9; 1.0	0.27	0.26	0.9;1.6
**SS**	0.05	0.03	0.6; 0.5	0.03	0.04	1.0; 1.9
**S**	0.18	0.15	0.8; 0.4	0.17	0.16	1.0; 1.8
**Blood systolic pressure**						
	n = 635	n = 100		n = 339	n = 81	
**SL**	0.24	0.32	1.6; 0.1	0.24	0.35	1.8; 0.06
**SS**	0.03	0.08	**2.9; 0.02**	0.03	0.06	2.6; 0.2
**S**	0.16	0.24	**1.7; 0.004** ^**A**^	0.15	0.23	**1.7; 0.02** ^**B**^
**Blood diastolic pressure**						
	n = 618	n = 117		n = 320	n = 100	
**SL**	0.24	0.32	1.6; 0.06	0.24	0.31	1.5; 0.2
**SS**	0.03	0.09	**3.4; 0.002**	0.02	0.08	**4.4; 0.004**
**S**	0.15	0.24	**1.8; 0.001** ^**C**^	0.14	0.24	**1.8; 0.004** ^**D**^

Comparisons were made relative to the LL genotype and L allele. Quantitative analysis, Beta value:

^**A**^ 1.6, *P* = 0.07.

^B^ 3.2, *P* = 0.009.

^**C**^1.3, *P* = 0.03,

^**D**^ = 1.7, *P* = 0.04.

*P* values were adjusted by age,

BMI, gender, medicament status, and Bonferroni correction.

After adjustment for age, gender, and BMI, we also observed a significant association of *NQO1* C609T SNP with low HDL-c level (OR = 7.7, *P* = 0.04) and hypertriglyceridemia (OR = 2.6, *P* = 0.04) but only among individuals suffering MetS. Either hypertriglyceridemia or HDL-c associations showed an additive model, with close of 4- and 1.5-fold higher than that displayed for the single risk allele, respectively. Quantitative analysis revealed significant differences only with low level of HDL-c (beta −1.8, *P* = 0.0008) (Table 5). No other components of MetS were associated with this SNP.

**Table 5:** Associations of *NQO1* C609T polymorphisms with components of metabolic syndrome.

	Total population			Metabolic syndrome		
	Below	Above	OR; *P*	Below	Above	OR; *P*
**Glucose**						
	n = 284	n = 451		n = 52	n = 368	
**CT**	0.52	0.53	1.0; 1.6	0.62	0.51	0.7; 0.5
**TT**	0.19	0.18	0.9; 1.2	0.13	0.17	1.0; 1.9
**T**	0.45	0.44	0.9; 1.2	0.44	0.43	0.9; 1.6
**HDL-C**						
	n = 573	n = 162		n = 389	n = 31	
**CT**	0.51	0.56	0.9; 1.2	0.52	0.55	1.34; 0.8
**TT**	0.19	0.16	1.1; 1.2	0.18	0.03	**7.7; 0.04**
**T**	0.45	0.44	1.0; 1.7	0.44	0.31	**1.9; 0.05** ^**a**^
**Triglycerides**						
	n = 286	n = 449		n = 73	n = 347	
**CT**	0.51	0.53	1.1; 1.0	0.51	0.53	1.4; 0.18
**TT**	0.19	0.18	1.0; 1.6	0.1	0.18	**2.6; 0.04**
**T**	0.44	0.45	1.0; 1.7	0.35	0.45	**1.6; 0.05**
**Waist Circumference**						
	n = 388	n = 347		n = 143	n = 277	
**CT**	0.51	0.54	1.1; 1.1	0.52	0.53	1.1; 1.4
**TT**	0.19	0.18	1.0; 1.7	0.16	0.17	1.1; 1.4
**T**	0.45	0.45	1.0; 1.8	0.42	0.44	1.1; 1.2
**Blood Systolic Pressure**						
	n = 635	n = 100		n = 339	n = 81	
**CT**	0.53	0.48	0.8; 0.9	0.54	0.48	0.8; 0.8
**TT**	0.18	0.21	1.1; 1.4	0.16	0.19	1.0; 1.8
**T**	0.44	0.45	1.0; 1.8	0.43	0.43	1.0; 1.8
**Blood Diastolic Pressure**						
	n = 618	n = 117		n = 320	n = 100	
**CT**	0.53	0.51	0.9; 1.4	0.53	0.51	0.9; 1.4
**TT**	0.18	0.18	0.9; 1.4	0.17	0.17	1.0; 1.8
**T**	0.45	0.44	0.9; 1.4	0.43	0.43	1.0; 1.9

Comparisons were made relative to the CC genotype and C allele.

^a^Quantitative analysis: beta = −1.8, *P* = 0.0008. *P* values were adjusted by age, BMI, gender, medicament status, and Bonferroni correction.

**Fig 1:**
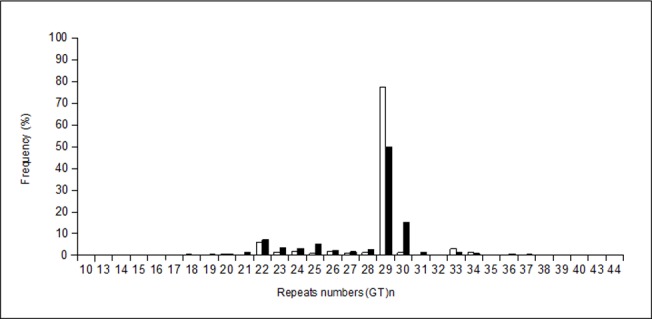
Frequency distribution of *HMOX1* (GT)*n* polymorphism. The number of repeats in Amerindian population (white bar, n = 241), and Mestizo population (black bar, n = 735) are shown as percentage.

On the other hand, when we compared the allele distributions between Amerindians and Mestizos *HMOX1* (GT)*n* repeat number distribution significantly differed between them. In both populations, the distribution showed peaks at 22 and 29 repeats; however, Amerindians showed a significantly higher frequency of 29 repeats (77.5% vs. 49.8%). Additionally, a third peak at 30 GT repeats was observed only in Mestizos (15%) (Fig 1). Significant differences in genotype and allele frequencies of *HMOX1* and *NQO1* between the two populations were also observed (*P* < 0.04, Table 6). In both Mestizos and Amerindians, the most common *HMOX1* genotype was homozygous LL, although the frequency of the S allele was significantly lower in Amerindian than Mestizo individuals (11% vs. 17%; *P* = 0.01; Table 6). Likewise, in both populations, the most common *NQO1* genotype was heterozygous CT, but the frequency of the risk allele significantly differed between the two groups (54% in Amerindians vs. 45% in Mestizos; *P* = 0.0008; Table 6).

**Table 6:** Genotype and allele frequencies of *HMOX1* and *NQO1* polymorphisms in Amerindian and Mestizo populations.

Gene	Populations		*P* value
	Amerindian n = 241	Mestizo n = 735	
*HMOX1*			
LL	0.8 (192)	0.71 (519)	
LS	0.18 (43)	0.25 (187)	
SS	0.02 (6)	0.04 (29)	**0.04**
L	0.89 (427)	0.83 (1225)	
S	0.11 (55)	0.17 (245)	**0.01**
*NQO1*			
*CC*	0.23 (56)	0.30 (215)	
*CT*	0.46 (111)	0.52 (385)	
*TT*	0.31 (74)	0.18 (135)	**0.0004**
*C*	0.46 (223)	0.55 (815)	
*T*	0.54 (259)	0.45 (655)	**0.0008**

Data are presented as frequency (sample size).

*P* values were adjusted by Bonferroni correction.

## Discussion

The *HMOX1* gene encodes the cytoprotective protein HO-1, which has important anti-oxidant and anti-inflammatory properties through its metabolites: biliverdin/bilirubin, carbon monoxide (CO), and free iron (Fe) [19–21]. Variations in the number of GT dinucleotide repeats in the promoter region of *HMOX1* can modulate the HO-1 transcription and protein levels [11,22]. The short allele (<25 GT dinucleotide repeats) is associated with increased gene expression and higher enzyme activity compared to the long allele (≥25 repeats) [23]. On the other hand, *NQO1* encodes a cytosolic antioxidant flavoprotein that catalyses the reduction of highly reactive quinone metabolites by using NADH. Studies of the C609T *NQO1* polymorphism have demonstrated that the T allele encodes a protein with a shorter half-life than the wild type, and that TT homozygotes have almost no NQO1 activity [24].

Polymorphisms in *HMOX1* and *NQO1* have been associated with several oxidative stress-related diseases, including cancer, diabetes, and Alzheimer’s disease [9,10,14–17]. MetS is an important public health issue. In recent years, the involvement of oxidative stress in the pathophysiology of this entity has been widely documented. Our present investigation of the involvement of *HMOX1* (GT)*n* and *NQO1* C609T polymorphisms in the susceptibility of MetS or its components within a Mexican population revealed that either the *HMOX1* (GT)*n* or *NQO1* C609T polymorphisms were associated with some of the MetS components but not with MetS. It was not surprising that there were only associations with MetS components, since MetS is indeed a syndrome with wide heterogeneous clinical expressivity and each one of its components is a complex trait itself.

*HMOX1* S allele was associated with high systolic and diastolic blood pressure, independently of MetS status. In accordance with these findings, a previous study reported an association of a polymorphism that also increased *HMOX1* expression (−413T/A) in Japanese women with hypertension [25]. The effect of HO-1 in hypertension seems to be mediated by CO production through HO-1-dependent degradation of the heme group. However, the role of CO in hypertension remains controversial since this may behave as a vasodilator that regulates vascular tone in blood vessels and thus reduces elevated systolic blood pressure, or as an endothelial dysfunction factor that promotes hypertension [20,26,27]. Otherwise, environmental exposure to inorganic arsenic (iAs) reportedly increases blood pressure [28], and exposure of human lymphoblastoid cells to iAs has been demonstrated to increase HO-1 levels [29]. Since hypertension is a complex disease, the impact of *HMOX1* (GT)*n* on hypertension may not be straightforward but could be modulated by environmental and other genetic factors. Supporting this concept, HO-1 induction in a salt-sensitive rat model contributes to salt-induces hypertension [30], and the pharmacological inhibition of HO-1 in obese Zucker rats decreases blood pressure [31].

Likewise, the *NQO1* 609T allele displayed an association with hypertriglyceridemia and low levels of HDL-c, but only within the group of individuals suffering MetS. In the same vein, a study of a population from India showed significantly increased triglyceride levels in TT homozygous individuals, but did not find any association with HDL-c levels [9]. Supporting these results, *nqo1*−/− mice exhibit higher triglyceride levels than their normal counterparts [32], whereas pharmacological activation of *nqo1* in mice models of MetS results in lower triglyceride levels [33]. Although the exact mechanism underlying the effect cytoprotective of *NQO1* is still unclear, it is know that ROS overproduction results in vascular dysfunction, which promotes the activity of adipocyte transcription factors leading to the dysregulated triglycerides and HDL-c synthesis [34].

On the other hand, the frequency of the *HMOX1* S allele in the Mestizo sample (17%) was one of the lowest observed worldwide, and an even lower frequency was found among the Amerindian population (11%), suggesting high heterogeneity of *HMOX1* S allele distribution among populations [17,35–37]. Our results in Mestizos are similar to those previously reported in a Hispanic population from the United States (15%) [38], but different from findings in populations from Chile (45%) [39] and Spain (32.6%) [35]. Surprisingly, a Mestizo population from the Occidental region of Mexico showed a higher frequency of the *HMOX1* S allele (25%) than that presently observed [40]. This discrepancy suggests that the S allele distribution may vary across the country. Since the Mexican population shows great genetic diversity with a south-to-north gradient of Amerindian ancestry, it is likely that the higher S allele frequency observed in the Occidental region might be influenced by a higher European genetic background in this Mexican land.

We observed over 28 different alleles of (GT)*n* in Mestizos and 19 alleles in Amerindians. Two peaks at 22 and 29 repeats in both Amerindian and Mestizos were observed, however we found an additional peak at 30 repeats in Mestizos (16%), which was almost non-existent in Amerindians. This distribution was similar to that reported in another study in a Mexican-Mestizo population [23]. The (GT)_30_ allele is the most frequent among Spaniards (40%) [35], others Caucasian [37,41,42] and Asian populations [43]. Conversely, the (GT)_29_ peak was highly frequent in Amerindian population (80%), but is rare or almost non-existent in other populations [35,37,41]. Ancestry analysis of random samples showed an average Amerindian ancestry of >90% for Amerindians and <59% for Mestizos. Thus, it is possible that the (GT)_30_ allele is a contribution from Caucasians, while the (GT)_29_ is an Amerindian contribution to Mexican Mestizos. Regarding *NQO1*, the frequencies of the 609T allele in Amerindians (54%) was the highest values reported worldwide. Our finding in Mestizos (45%) was similar to that reported in another study of asthmatic children form Mexico City (44%) as well as those observed in studies of Asian (41.9%) and Indian (42.46%) individuals [9,44,45], nevertheless, it was higher than those previously observed in Caucasians (14–18%), Iberians (18%), and Yorubas (18%) [15,45,46].

## Conclusions

The present study provides additional evidence that polymorphisms of the *HMOX1* and *NQO1* genes are associated with high risk in metabolic disorders, such as blood systolic and diastolic pressure, hypertriglyceridemia, and low levels of HDL-c. These findings suggest that the frequency of *HMOX1 and NQO1* alleles in Mexican Mestizos results from the admixture between Caucasian and Amerindian populations. Further studies are necessary to confirm these data.
